# Progress in climate modeling of precipitation over the Tibetan Plateau

**DOI:** 10.1093/nsr/nwaa006

**Published:** 2020-02-03

**Authors:** Qing Bao, Jian Li

**Affiliations:** 1 State Key Laboratory of Numerical Modeling for Atmospheric Sciences and Geophysical Fluid Dynamics, Institute of Atmospheric Physics, Chinese Academy of Sciences, China; 2 State Key Laboratory of Severe Weather, Chinese Academy of Meteorological Sciences, China

Given the controversy regarding the ‘Monsoon Melee’, there is a need for more accurate Tibetan Plateau (TP) climate modeling that clarifies its role in monsoon variations [1]. Because of the unique geographical characteristics of the region, TP climate modeling faces many challenges. One of the largest modeling biases is the overestimation of precipitation along the southern slopes of the TP, which influences the simulation of the monsoon circulation through the teleconnection associated with the local latent heat flux. This also affects estimates of landslide risk and water resources. Recent advances in climate modeling of convection, land–atmospheric interaction and large-scale dynamics can mitigate the simulated biases associated with the TP and, in turn, improve disaster-mitigation planning in this region.

**Figure 1.**
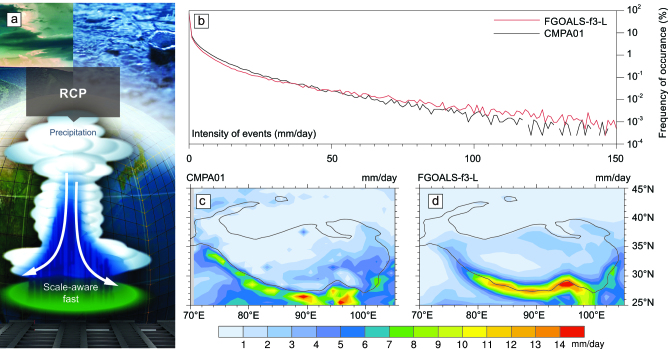
(a) Schematic representation of the Resolving Convective Precipitation (RCP) scheme (adapted from the cover of [3]). (b) Probability distribution of daily precipitation; (c) and (d) geographic distribution of boreal summer precipitation over the TP. The FGOALS-f3-L data were obtained from the CMIP6 ESGF nodes [3] and the CMPA01 data are based on a high-spatio-temporal-resolution analysis of merged gauge and satellite precipitation observations.

Physics-based climate-model parameterizations have been developed to reduce the biases associated with TP climate modeling. Bao *et al.* [2] and He *et al.* [3] developed a Resolving Convective Precipitation (RCP) scheme within a low-resolution version of the Flexible Global Ocean–Atmosphere–Land System model, finite-volume version 3 (FGOALS-f3-L), and applied this to the Model Intercomparison Projects as well as real-time climate-prediction projects. The RCP scheme has the advantage of both scale-awareness and high computational efficiency. In contrast to conventional convective parameterization, the RCP scheme calculates convective and stratiform precipitation explicitly at the grid scale (Fig. 1a). As illustrated in Fig. 1b–d, FGOALS-f3-L successfully minimizes the overestimation of precipitation on the southern slopes of the TP and accurately reproduces the probability distribution of TP daily precipitation, particularly for extreme precipitation that exceeds 100 mm/day. With respect to land–atmosphere interactions, Lee *et al.* [4] developed a Monte Carlo 3-D radiational approach and applied this to climate modeling. This approach uses a new land-surface configuration consisting of more realistic 3D triangles that replace the previous stair-like rectangles. Their results revealed that the 3D topography reduces the bias associated with the TP land–atmosphere flux and helps to resolve the problem of overestimated precipitation on the TP.

Using super-high-resolution methods and a nesting solution with improved dynamic cores on state-of-the-art supercomputers, climate models can now generate more realistic representations of the thermal and dynamic effects of the TP. The Finite-Volume Cubed-Sphere Dynamical Core (FV3) is a scalable and flexible dynamic core. Zhou *et al.* [5] found that a stretched FV3 grid can accurately and efficiently reproduce the orographic effect on precipitation. Li *et al.* [6] noted that the distribution of rainfall over and around the TP and other high mountains becomes more realistic as the resolution increases, but the maximum bias also increases. By replacing the semi-Lagrangian method with a finite-difference approach in the trace–transport algorithm, Yu *et al.* [7] found that the simulation of precipitation over the steep southern margin of the TP was significantly improved, and they attributed this to the elimination of multi-grid water-vapor transport. The above breakthroughs in model physics parameterizations and dynamic core studies mitigate the problem of modeling precipitation over the TP, and benefit related fields such as disaster risk reduction and water-resource utilization on the Tibetan Plateau and surrounding areas.
